# Affective Prosody and Its Impact on the Neurology of Language, Depression, Memory and Emotions

**DOI:** 10.3390/brainsci13111572

**Published:** 2023-11-09

**Authors:** Elliott D. Ross

**Affiliations:** 1 Department of Neurology, University of Oklahoma Health Science Center, Oklahoma City, OK 73104, USA; elliott-ross@ouhsc.edu or elliott.ross@cuanschutz.edu; 2 Department of Neurology, University of Colorado School of Medicine, Aurora, CO 80045, USA

**Keywords:** acoustical phonetics, affective prosody, aphasia, aprosodia, melancholic depression, non-melancholic depression, primary emotions, social emotions

## Abstract

Based on the seminal publications of Paul Broca and Carl Wernicke who established that aphasic syndromes (disorders of the verbal–linguistic aspects of communication) were predominantly the result of focal left-hemisphere lesions, “language” is traditionally viewed as a lateralized function of the left hemisphere. This, in turn, has diminished and delayed the acceptance that the right hemisphere also has a vital role in language, specifically in modulating affective prosody, which is essential for communication competency and psychosocial well-being. Focal lesions of the right hemisphere may result in disorders of affective prosody (aprosodic syndromes) that are functionally and anatomically analogous to the aphasic syndromes that occur following focal left-hemisphere lesions. This paper will review the deductive research published over the last four decades that has elucidated the neurology of affective prosody which, in turn, has led to a more complete and nuanced understanding of the neurology of language, depression, emotions and memory. In addition, the paper will also present the serendipitous clinical observations (inductive research) and fortuitous inter-disciplinary collaborations that were crucial in guiding and developing the deductive research processes that culminated in the concept that primary emotions and related display behaviors are a lateralized function of the right hemisphere and social emotions, and related display behaviors are a lateralized function of the left hemisphere.

## 1. Introduction

This publication will review, in detail, the neurological research establishing that language is a distributed bi-hemispheric function. The main focus will be on the right hemisphere’s contribution to language and the research process that led to the development of a nascent neurology of depression and to a more nuanced understanding of the neurologic basis of memory and emotions. In addition, the serendipitous clinical observations and fortuitous inter-disciplinary collaborations that were crucial factors in motivating the deductive research process will be presented to emphasize that scientific advances are most often the result of inductive rather than deductive research [1,2,3,4] (note: Table 1 in reference [1] was printed incorrectly; see Table 3 in reference [5] for a correct rendition). Inductive research is a bottom-up process that is usually initiated by unexpected, often unique, clinical observations [6,7]. This, in turn, generates a tentative hypothesis and eventually a sustainable theory, if it receives support through deductive research. In contrast, deductive research is a top-down process that usually begins with a theory, not necessarily based on hard facts or data, that leads to hypothesis testing through controlled research paradigms to confirm or negate the hypothesis. Since this publication will highlight how the inductive–deductive research process unfolded over time, it is best characterized as a narrative, rather than a traditional, scientific review.

## 2. Neurology of Language

### 2.1. The Aphasias

The modern discipline of behavioral neurology and cerebral localization of complex brain functions was initiated by Paul Broca in 1861 [8] when he reported a patient who had loss of articulate speech with relative preservation of comprehension. On autopsy, the patient had suffered a progressive lesion that destroyed the left posterior portion of the third frontal gyrus. It should be noted that the autopsy did not include sectioning of the brain; however, a CT scan of the preserved brain has shown that the lesion also extended into the insula, deep white matter and striatum [9]. He called this syndrome “aphemia” that was later changed by others to Broca or motor aphasia. In a subsequent publication, Broca [10] reported that lesions involving the right as opposed to the left frontal operculum did not result in aphemia, thus establishing that articulate speech was most likely a lateralized function of the left hemisphere.

**Figure 1:**
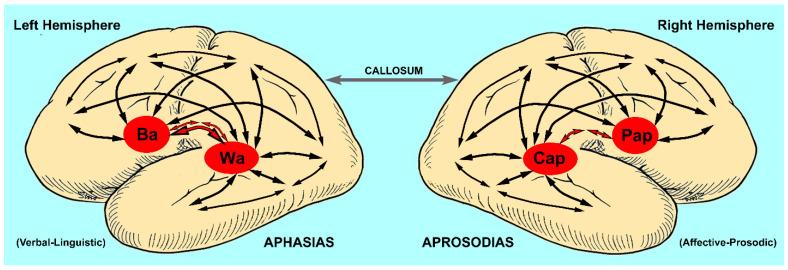
Cortical map for the neuroanatomical basis of language (Ba = Broca’s area or motor speech center, Wa = Wernicke’s area or acoustic speech center, Cap = comprehension of affective prosody or acoustic affect center, Pap = production of affective prosody or motor affect center, red-filled arrows = arcuate fasciculus). Note that the arcuate fasciculus is anatomically different for each hemisphere. The left arcuate fasciculus is composed of a direct bundle and an indirect bundle that synapses in the inferior parietal lobe, whereas the right arcuate fasciculus is composed mainly of an indirect bundle that synapses in the inferior parietal lobe with a mostly vestigial direct bundle [29,30]. Comprehension and production of language is accomplished through various distributed parallel neural networks with Broca’s and Wernicke’s areas and their analogs in the right hemisphere (Pap, Cap), serving as nodal points in conjunction with modality specific (unimodal) and higher-order (heteromodal) associational regions of the neocortex (multiple black arrows) [31]. Lesions that injure the peri-Sylvian (opercula) cortex cause aphasias and aprosodias that have, in common, loss of repetition (motor, sensory, conduction and global syndromes). Lesions that injure the para-Sylvian associational regions of the neocortex cause aphasias and aprosodias that have, in common, preservation of repetition (transcortical motor, transcortical sensory, mixed transcortical and anomic or agesic syndromes) [15]. The frontal motor-related language regions of each hemisphere interact via the anterior corpus callosum to pre-condition each other before each hemisphere generates its motor outputs to the brainstem for the production of articulate speech to ensure that the verbal–linguistic and affective prosodic components are temporally and behaviorally coherent (see Section 2.4, fourth and last paragraphs) [32,33]. Not included in the map are subcortical structures that are part of an expanded neural network underlying language [15].

Starting in 1874, Carl Wernicke published a series of articles (translated and annotated by Eggert [11]), in which he described patients with lesions involving the posterior temporal operculum of the left hemisphere who had lost their ability to comprehend speech. In contrast to patients with Broca aphasia, their speech was fluent and well-articulated but often unintelligible because of paraphasic errors (incorrect word choices that were either semantically or phonologically close to the intended word or pure neologisms), i.e., Wernicke or sensory aphasia. Like Broca, Wernicke also reported that lesions of the right hemisphere, specifically involving the posterior temporal opercula region, did not result in sensory aphasia, thus reinforcing the concept that the verbal–linguistic aspects of language are a lateralized function of the left hemisphere. In addition, he proposed that language was represented in the left hemisphere as a large-scale distributed cortical network with “Broca’s” area (motor speech center) serving as the nodal point for speech production, “Wernicke’s” area (acoustic speech center) serving as the nodal point for verbal comprehension and the surrounding, non-opercula neocortex cortex serving as an “ideational” or “imagery” area in which vast repertoires of information are stored as memory of events, objects and people based on their sensory and motor attributes (see Figure 1, left panel) [4,11,12,13,14,15]. Contrary to traditional dictum, Wernicke did not localize comprehension to the acoustic speech center but thought that the role of the acoustic speech center was to extract from auditory space those sounds associated with language and to develop and store a phonemic representation of those sounds. Verbal comprehension occurs when the phonemic representations are matched, via parallel distributed neural connections, with the various imagery–ideational areas that store one’s life experiences. In a similar fashion, Wernicke did not localize articulate speech to Broca’s area. Since speech is a highly over-learned motor function, he reasoned that the motor speech center was critical for developing and storing the phonological code underlying articulation in conjunction with the motor and aforementioned sensory imagery–ideational areas. Lastly, he hypothesized that verbal repetition was accomplished via direct connections between the acoustic speech center and the motor speech center that he eventually attributed to the arcuate fasciculus [16]. Based on his neural network model of language, Wernicke predicted the existence of various aphasic syndromes that had not yet been described in the literature, in particular conduction aphasia, caused by lesions that disconnect the acoustic and motor speech centers, and aphasias in which the ability to repeat was preserved, caused by lesions that either disconnect the imagery–ideational areas from the acoustic and motor speech centers or injured the imagery–ideational areas directly, leaving intact the acoustic and motor speech centers and their connections via the arcuate fasciculus. Based on Wernicke’s concepts, Lichtheim [17] described various aphasic patients who had preserved repetition due to lesions outside the opercular speech centers. These syndromes were ultimately classified as “transcortical” types of aphasias [15,18,19]. Thus, by the close of the 19th century, eight distinct aphasic syndromes had been identified, four with relatively impaired repetition, Broca (motor), Wernicke (sensory), conduction and global, and four with relatively preserved repetition, transcortical motor, transcortical sensory, anomic and mixed transcortical or isolation aphasia (Table 1), and also the syndrome of pure word deafness. In the late 1800s and early 1900s, numerous papers were published to define the functional–anatomic localization of the various aphasic syndromes and whether or not lesions involving deep structures, such as the basal ganglia, were contributory or essential to the aphasic syndromes [15]. Recovery of aphasic deficits after acute focal left-hemisphere lesions was also observed by clinicians, making functional–anatomic correlations, at times, contentious. Nevertheless, the classic functional–anatomic correlations are reasonably accurate if the pathological lesion is acute and relatively localized, such as an ischemic stroke, and the examination is performed between two to six weeks post-stoke after acute injury effects, such as diaschisis and edema, abate and before the onset of long-term recovery [15,19,20,21]. Aphasias may occur after either cortical or deep lesions bringing into question that language is a strictly cortical function [15,22,23,24,25,26]. Deep lesions tend to be associated with transcortical aphasias and have relatively good potential for recovery. Small cortical lesions that injure either Broca’s area or Wernicke’s area are also associated with the rapid and relatively good recovery of function [15,27,28]. However, if the lesion is large, involving both cortical and deep structures, then recovery is usually limited [20].

Based on this brief summary of research involving the aphasias, language, in general, has been deemed a dominant and lateralized function of the left hemisphere. This, in turn, has led to the left hemisphere being characterized cognitively as the “major” hemisphere, with the right hemisphere being demoted to the status of the “minor” hemisphere [34], thus conceptually diminishing any role the right hemisphere may have in language and related behaviors. It should be noted, however, that Hughlings Jackson in the late 1870s [35,36,37] published a lengthy three-part article titled “On affections of speech from diseases of the brain” and observed that densely aphasic patients often tried to communicate their intentions through excessive gesticulations and emotional verbalizations (affective prosody), using whatever words they could articulate that often resulted in outright cursing. He also stated that language should be divided into two components, “Intellectual” or propositional (use of words and syntactical relationships to convey intentions) and “Emotional” (see p. 311 [35]), and induced, based on his clinical observations, that the emotional aspects of language and communication were, most likely, a right-hemisphere function. Yet, Jackson’s inductive observations regarding the potential role of the right hemisphere in language and communication were not investigated by clinicians for nearly 100 years, commencing with the publication by Heilman, Scoles and Watson [38] and subsequent publications by Tucker, Watson and Heilman [39], Ross and Mesulam [40] and Ross [41].

### 2.2. The Aprosodias

In 1975, Heilman, Scoles and Watson [38] published an intriguing article titled “Auditory affective agnosia. Disturbed comprehension of affective speech”. Six right-handed patients with right-sided lesions who had clinical neglect and six right-handed patients with left-sided lesions who had either conduction or anomic aphasia were assessed on their ability to identify the verbal–linguistic or the emotional content of sentences that were semantically neutral but spoken with either a happy, sad, angry, or indifferent affect. The patients had lesions involving, at minimum, the lateral parietal region based on clinical examination. On the verbal–linguistic portion of the assessment, both right- and left-brain-damaged (RBD, LBD) patients performed normally. In contrast, the RBD patients scored at chance when identifying the emotional content of the sentences whereas the LBD patients performed much better than chance. The difference in performance between patient groups was statistically significant and, behaviorally, extremely robust with an *r*^2^ effect size of 0.83 (calculated from the reported *t*-value and degrees of freedom [42]), explaining approximately 83% of the data variance. In a subsequent study, Tucker, Watson and Heilman [39] tested 11 right-handed patients with RBD and neglect and 7 right-handed patients with LBD and conduction aphasia with, at minimum, lateral parietal lesions based on clinical examination to identify and discriminate the emotional content of semantically neutral sentences. On the identification task, the RBD group performed at chance compared with the LBD group. The difference between groups was statistically significant and highly robust with an *r*^2^ of 0.76, as calculated from the reported *t*-value and degrees of freedom [42]. For the emotional discrimination task, the RBD group performed at chance compared with the LBD group. The difference between groups was extremely robust with a *r*^2^ of 0.90, as calculated from the reported *t*-value and degrees of freedom [42]. In the final part of the study, eight RBD patients with neglect and eight controls were assessed on their ability to repeat a neutral sentence, when asked to insert a happy, angry, sad, or indifferent emotion. Their performance was rated by three judges. Once again, the RBD group performed at chance compared with the controls. The difference was statistically significant and reasonably robust with an *r*^2^ of 0.52 [42]. Thus, over a two-year period, these two deductive research studies established that comprehension of emotional prosody was most likely a lateralized function of the right hemisphere. Although not reported in the papers, the patient (index case) that initiated the aforementioned deductive research was a woman who had an abscess involving the right posterior opercular region [43]. After she was fully treated and cured of the abscess, she and her husband returned to the clinic and her husband reported that they were experiencing marital difficulties and no longer had a “meaningful relationship”. When the patient was examined, it was discovered that she was not able to comprehend emotional prosody. Thus, she interpreted what was said to her literally rather than figuratively, causing loss of language competency and psychosocial difficulties.

The next incremental development was published by Ross and Mesulam in 1979 [40]. (Note: Marsel Mesulam and I did our Neurology residencies together at the Boston City Hospital under the guidance of Prof. Norman Geschwind). We presented two right-handed patients who lost their ability to project emotion in their voice and gestures either spontaneously, on request, or when asked to imitate the examiner’s voice and facial expressions. 

The first patient was a school teacher who was admitted at Parkland Memorial Hospital (Dallas, TX, USA) because of the acute onset of a left hemiplegia with somatosensory loss that rapidly improved. On CT scan, she had an ischemic infarction involving the right supra-Sylvian opercula cortex and underlying white matter that spared the basal ganglia. When she was in the hospital, I met her husband and two sons, who seemed quite devoted to her [1]. However, during the rounds, I found her in bed with a younger man. The younger man promptly sprang out of the bed and was clearly embarrassed. The patient, in a flat monotone voice, explained to me that she no longer loved her husband and was going to divorce him and marry her “boyfriend”, who was an English college professor. Over the next few days, I tried to counsel her regarding her condition and her decision to divorce her husband but found the interactions very trying because she did not seem emotionally invested in the conversations. On a follow-up clinic visit four weeks after discharge, she reported that “something is terribly wrong with me”. Since her stroke she could no longer control her classroom or discipline her students, which she attributed to loss of the ability to insert anger into her voice and actions to signal to them that “I meant business”. At home, however, she was better able to discipline her children since she would use curse words to obtain their attention. She also commented that she did not use curse words in her classroom because she thought it was unethical. Approximately six months after her stroke, she regained the ability to express emotion into her voice and demeanor to the point that she was once again able to control her classroom. 

The second patient was a retired surgeon who suffered a stroke that resulted in a dense left hemiplegia with sensory loss. Over several years, his hemiplegia gradually improved. He was seen in the Behavioral Neurology Clinic (Beth Israel Hospital, Boston) because his wife was seeking a divorce. His wife reported that after his stroke his personality had changed, resulting in a “nasty temperament”. She noted that when he asked her to do something, she perceived it as an outright demand rather than a solicitous request. The patient, however, thought that his communicative difficulties were due to his marked inability to vocally express emotions. On examination five years post-stoke, he had a moderate left hemiparesis and his speech was monotonic with a marked paucity of gestural behavior resulting in a flat affect. A CT scan demonstrated a large right supra-Sylvian infarction that also involved the basal ganglia, perhaps explaining why his flat affect had not substantially improved, in contrast to the school teacher reported above.

Both patients, based on their own observations and confirmed by bedside testing, were able to comprehend vocal and gestural displays of affect by others and reported that the ability to feel emotions inwardly despite their flat affect was intact. Based on the publications by Heilman and colleagues [38,39] that lesions of the right parietal region resulted in loss of the ability to comprehend vocal emotions and our observation that production of affective prosody is impaired by lesions involving the anterior mid supra-Sylvian region of the right hemisphere, we inductively reasoned that the functional–anatomic organization of affective prosody in the right hemisphere appeared to be analogous to the functional–anatomic organization of propositional language in the left hemisphere. Based on this hypothesis, I initiated a bedside clinical study that was published in 1981 [41].

Ten right-handed patients with acute focal RBD, localized by CT scan, were assessed for affective prosodic deficits using a bedside examination similar to the bedside examination for aphasic deficits. Spontaneous affective prosody and facial expressions were rated during an interview that included asking the patient to recall emotional life events. In addition, the ability to repeat a neutral sentence using different emotions (happy, sad, angry, indifferent) and the ability to comprehend affective prosody and facial expressions of emotion were also assessed. Depending on lesion location, different combinations of affective communication deficits were observed that could be readily classified into syndromes that were analogous to aphasic deficits after focal LBD. Thus, the term “aprosodia” (ā′ pro **sō**′ dia) was coined and different adjective modifiers were used to classify the syndromes (Table 1). Three cases of motor aprosodia were identified who had CT-scan-verified infarctions involving the right anterior supra-Sylvian region. One patient had a relatively small lesion that spared the basal ganglia and had a rapid improvement of his motor aprosodia over the course of two months. The other two patients had lesions that also involved the basal ganglia and did not show rapid clinical improvement. One patient had sensory aprosodia due to an ischemic lesion involving the posterior parietal and posterior temporal operculum. His ability to comprehend facial expressions moderately improved over the course of two weeks whereas his ability to comprehend affective prosody remained severely impaired. One patient had global aprosodia due to an ischemic infarction involving the entire middle cerebral artery distribution that also involved the basal ganglia. One patient had transcortical sensory aprosodia due to a small hematoma in the anterior mid temporal lobe that spared the posterior superior temporal opercular region. One patient had mixed transcortical (isolation) aprosodia. The patient was first examined six months after undergoing surgical clipping of a cerebral aneurysm that resulted in a large supra-Sylvian infarction that extended into the posterior superior temporal gyrus and basal ganglia. Her affective–prosodic repetition was relatively preserved compared with her spontaneous affective prosody and her ability to comprehend affective prosody. Based on the lesion localization, most likely she had an initial global aprosodia that, over time, recovered to a transcortical motor aprosodia. One patient was classified as having motor aprosodia with pure affective deafness. The lesion on CT scan showed two distinct infarctions, one involving the frontal operculum and anterior insula, accounting for his motor aprosodia and the other involving the right anterior and mid temporal operculum most likely accounting for his pure affective deafness.

In a subsequent study using CT scan localization, Gorelick and Ross [44] reported on 14 right-handed patients with acute RBD, mostly from ischemic infractions and a few from small intracerebral hemorrhages, that corroborated the existence of various aprosodia syndromes and their functional–anatomic associations, as originally reported by Ross [41]. In addition, recovery of affective–prosodic deficits over time was documented where an aprosodic syndrome transitioned into another type of aprosodia that was usually due to improvements in affective–prosodic repetition. The patients were collected consecutively over a six-month time span by Gorelick during his stroke fellowship at Michael Reese Hospital and Medical Center (Chicago, IL, USA). Twelve patients were diagnosed with aprosodia: six with motor, one with global, two with conduction, one with sensory, one with transcortical sensory and one with pure affective deafness. Two patients did not have any affective–prosodic deficits on examination: one with a small hemorrhage involving the lateral thalamus that spared the basal ganglia and the other with a lesion involving the inferior occipital lobe that spared the splenium of the corpus callosum and the angular gyrus. One of the most interesting findings was that over the same six-month time span, Gorelick had concurrently evaluated 15 patients with LBD. Twelve patients were found to have an aphasic syndrome, thus establishing that the aprosodias were not esoteric, rarely encountered, clinical syndromes but that they occur as commonly as the aphasias, if clinicians are willing to actively assess for affective–prosodic deficits.

The only missing syndrome from the above two clinical studies to complete the concept that the aprosodic syndromes are analogous to aphasic syndromes was a case of “Anomic” aprosodia (loss of the ability to identify facial expressions with intact comprehension of affective prosody, affective–prosodic repetition and spontaneous affective prosody and facial expressions). However, Bowers and Heilman [45] published a case report of a patient with a right-hemisphere tumor located in the deep white matter underlying the angular gyrus region that was consistent with the concept of “anomic” aprosodia. This syndrome has been termed agesic aprosodia because the patient was not truly anomic except for his inability in naming (and identifying) facial expressions (Table 1).

### 2.3. Subcategories of Prosody and Their Acoustic Signatures

Prosody, in general, is considered a paralinguistic aspect of language [46] that can be divided into three major classifications [47,48]: intrinsic, intellectual and emotional [49,50]. Intrinsic prosody is used for linguistic purposes to help clarify the verbal–linguistic aspects of discourse. Phonemic or lexical stress can alter the meaning of a word, especially if spoken in isolation. For example, “con-**tract**” (noun) versus “**con**-tract” (verb) or “green **house**” (green colored house; adjective-noun) versus “the **green**-house” (noun). Prosodic (contrastive, emphatic) stress of a word may alter sentential meaning; for example: “**I** did not take the book yesterday” (implying that someone else did) versus “I did **not** take the book yesterday” (outright denial) versus “I did not take the book **yesterday**” (implying some other day). Prosodic stress may also help keep track of dialogue during discourse (pragmatics); for example: “**What** is Bob bringing to the party?”—”He’s bringing the **salsa**”. Lastly, the use of prosodic stress and pauses between words may help clarify potentially ambiguous syntax [51]. For example: “Danny wrote … to his **friend** from Dallas” versus “Danny wrote to his friend … from **Dallas**”. Intellectual prosody inserts attitudes into discourse such as sarcasm, disbelief, affirmation and irony [52]. For example, if one states that “Jill is beautiful” with strong prosodic stress on “**is**”, it is an affirmation of Jill’s beauty in agreement with others. However, if strong prosodic stress is placed on “**beautiful**” with a rising (questioning) intonation, it signals disbelief with others. When individuals are presented with statements in which the attitudinal prosody (figurative meaning) is in disagreement with the verbal–linguistic message (literal meaning), they will overwhelmingly interpret the statement based on the attitudinal prosody [53,54,55,56], thus emphasizing the corollary that “how one says something is often more important than what is actually said” [42]. Emotional prosody is the insertion of raw emotional intent into discourse. The term affective prosody combines intellectual and emotional prosody into a single category [49]. When patients lose the ability to comprehend and/or properly produce affective prosody, they lose communication competency that, in turn, may lead to loss of psychosocial well-being and disruption of social interactions.

The acoustic signatures of prosody involve the dynamic manipulation of various acoustical features associated with fluent articulation: overall pitch, manipulation of pitch over time (intonation), loudness, syllable and word durations, pauses between words, timbre, vowel quality and tempo [50]. In English, a non-tone language, prosodic stress has been traditionally attributed to a brief rise and fall in pitch, termed pitch obtrusions [57,58]. However, recent research in normal controls and patients with focal brain damage has demonstrated that prosodic stress, similar to lexical stress, is signaled by changes in pitch, loudness and/or duration through complex trading and linguistic conditioning effects [59] that are often idiosyncratic to an individual [50]. Affective prosody, however, has been shown to rely mainly on the ability to vary pitch over time, producing intonation contours in speakers of non-tone languages, such as English, a subject that will be addressed in detail in Section 2.4 [60,61,62]. Pitch is produced during the voiced portions of articulation due to vibrations arising from the vocal folds. Voiced articulations occur during the production of all vowels and some parts of consonants. Take the word “trace” as an example. It is composed of two consonants (“tr” and “ce”) and a vowel (“a”). The “t” is an unvoiced plosive, the “r” and “a” are voiced and the “ce” is an unvoiced sibilant. When the vocal folds vibrate, they produce a fundamental frequency (F_0_) and various harmonics that are acoustic multiples of musical octaves. To illustrate, if F_0_ is 100 Hz, then the first harmonic is 200 Hz (one octave above), the second harmonic is 400 Hz (two octaves above) and the third harmonic is 800 Hz (three octaves above). The F_0_ and harmonics are enhanced or attenuated by the vocal tract (areas above the vocal folds) over time to produce various vowels and parts of consonants [63,64,65].

In 1981, Voice Identification Inc. (Manville, NJ, USA) developed the PM Pitch Analyzer that was able to track changes in F_0_ in Hz and loudness in decibels (dB) over time and displayed the results on a cathode ray tube. Using programmable cursors to mark the start and end of a spoken phrase or utterance, the Pitch Analyzer extracted and displayed the Hz and dB means and standard deviations (SD). In 1982, I was able to acquire the Pitch Analyzer through funds generously provided to me by the Stuttering Foundation, Baylor College of Medicine, Houston, TX (established by David Rosenfield, MD; a longtime friend and behavioral neurologist who I met in 1975 at the Aphasia Unit, Boston VA Hospital, when he was a Fellow on the Unit and I was doing a rotation as part of my neurology residency), and through the encouragement of Frances Freeman, PhD (Assoc. Prof., Callier Center, University of Texas at Dallas, who David Rosenfield suggested that I contact for possible collaborative research) [1]. Frances also introduced me to the science of acoustical phonetics ([63,64,65]; see above paragraph) and suggested that measuring F_0_ dynamics might be a good way to quantitatively assess production of affective prosody [1]. The initial preliminary research study using the Pitch Analyzer was presented at a poster session at the 1983 American Academy of Neurology Meeting in San Diego, CA [60]. Eight right-handed patients with ischemic infarctions confirmed by CT scan and six age-equivalent healthy controls were tested and tape recorded on their ability to produce spontaneous affective prosody, by asking them to recall a past emotional experience, and on a repetition task in which they were asked to mimic the sentence “I am going to the movies” using neutral, angry, sad, surprised and happy intonations that were presented to them using stimuli recorded on an audio tape. The patient group consisted of two patients with Broca aphasia, two patients with motor aprosodia, two patients with Wernicke aphasia and two patients with sensory aprosodia. The subjects’ taped utterances were analyzed using the Pitch Analyzer and, based on the Hz means and standard deviations, a percent co-efficient of variation (F_0_CV%) was calculated for each utterance. The only statistically significant findings when comparing patients to controls was that patients with motor aprosodia had markedly reduced average F_0_-CV% for both spontaneous affective prosody [Z-score (based on control performance) of −3.55 and −3.23] and affective–prosodic repetition [Z-scores of −3.43 and −3.93]. In comparison, patients with Broca and Wernicke aphasia and sensory aprosodia performed no differently from controls on either task. However, one patient with sensory aprosodia had a markedly reduced F_0_-CV% on affective repetition (Z-score of −2.93), while the other did not (Z-score of −0.68), thus no statistically significant differences were found when compared with controls, an issue that will be addressed in Section 2.4 (fourth paragraph).

In 1985, Shapiro and Danly [61] assessed 16 right-handed patients with ischemic or hemorrhagic lesions localized to either the right or left hemispheres and five age-equivalent neurologically healthy controls on their ability to manipulate their F_0_ on various reading tasks while intoning their voices in either a declarative or interrogative mode (non-emotional prosody) versus a happy or sad mode. The subjects’ responses were tape recorded and each target sentence was analyzed based on seven F_0_ measures using Henke’s Fundamental Period program (developed at the Research Laboratory of Electronics, Massachusetts Institute of Technology) that was executed on a PDP-9 laboratory computer (Digital Equipment Corporation, Maynard, MA, USA). The patients were subcategorized, based on their CT scans, into those with right anterior (pre-Rolandic), right central (pre- and post-Rolandic), right posterior (post-Rolandic) and left posterior brain damage. They found that patients with right anterior and right central brain damage had markedly less pitch variation with a restricted F_0_ range on both the emotional and non-emotional intonational tasks compared to patients with left posterior brain damage and controls. In addition, they reported that patients with posterior RBD had exaggerated intonational range on both the non-emotional and emotional intonational tasks compared with controls.

In 1986, I was able to purchase from Heathkit (Inc., Benton Harbor, MI, USA) a PDP LSI 11/23 (Digital Equipment Corporation) desk-top computer with a grand total of 128 kilobytes of memory and interfaced it with the PM Pitch analyzer using a 16-bit parallel interface. This allowed me to write a dedicated computer program using Basic (Microsoft, Inc., Redmond, WA, USA) that took the digitized Hz and dB data generated by the Pitch Analyzer and calculated 13 statistical measures involving F_0_, dB and time-related metrics to acoustically assess affective prosody (see reference [62] for a detailed mathematical description of how the measures were developed for quantitively assessing affective–prosodic production in tone and non-tone languages). The F_0_ Hz data were converted into semitones, a logarithmic, interval-preserving, pitch scale in which octave changes are equal to 12 semitones. This is necessary because if pitch changes are recorded in Hz, then subjects with higher natural pitch ranges, such as women and children, cannot be statistically compared with subjects with lower natural pitch ranges, such as adult men. The dB data did not need converting since it is an interval-preserving, logarithmic scale. There were five semitone measures related to F_0_: register (mean semitones across the utterance), slope, variation (standard deviation of semitones across the utterance), attack (average velocity of semitone variability across the utterance) and delta (difference in pitch contour compared with an affectively neutral utterance), and five dB measures related to loudness: register (mean dB across the utterance), slope, variation (standard deviation of dB across the utterance), attack (average velocity of dB variability across the utterance) and delta (difference in dB contour compared with an affectively neutral utterance). In addition, the total time of the utterance was calculated as a log_10_ function and a % voicelessness time and a % pause time were calculated for the utterance under analysis.

### 2.4. Acoustic Realization of Affective Prosody in Tone and Non-Tone Languages

In 1982, I fortuitously met Jerold Edmondson, PhD (Professor of Linguistics, Department of Foreign Languages, University of Texas at Arlington), at a Linguistic Forum Series hosted by the International Linguistic Center in Dallas, where he presented his research on tone languages and I presented an overview of the roles of the left and right hemispheres in language and communication. He was very interested in the research we were performing to establish that deficits in affective–prosodic production in RBD patients were most likely the result of the loss of the ability to manipulate F_0_ across time [60]. He then introduced me to the acoustical phonetics underlying tone languages, and we decided to engage in collaborative research.

Languages can be dichotomized into tone versus non-tone [42,66]. Approximately half the world’s population, mainly residing in southeast Asia (China, Taiwan, Vietnam and Thailand) and sub-Saharan Africa, speak tone languages that use brief intonation contours called lexical tones when articulating words that are crucial for word meaning. For example, in Mandarin Chinese, if “ma” is articulated using a high flat tone, it means “mother”. If it is articulated with a rising tone, depending on context, it means either “numb”, “hemp” or “cannabis”. If it is articulated with a fall-rising tone, it means “horse”, and if it is articulated with a falling tone, it is interpreted as an upbraiding. Non-tone languages, such as English and various Indo-European romance languages, do not employ lexical tones for word meaning. For example, no matter how one intonates “ma” in English it always semantically understood as mother although the intonation may alter affective intent. In 1983, Hughes, Chan and Su [67] published a clinical study involving 19 subjects whose primary language was Mandarin Chinese, 12 with focal RBD and 7 controls. The subjects were evaluated on their ability to express, repeat, comprehend and discriminate affective prosody, comprehend lexical tones and comprehend and produce emotional gestures. Eleven of the twelve RBD patients were found to have aprosodic deficits when compared with the controls: three with sensory, two with motor, three with global and one with mixed transcortical aprosodias. The lesion locations observed on CT scan were, for the most part, consistent with those reported previously [40,41,44]. Although the RBD patients were slightly impaired in comprehending lexical tones when compared with controls, the difference was not statistically significant and none of the RBD patients were found to be clinically aphasic on formal testing. These findings suggested the following: (1) the modulation of lexical tones is a verbal–linguistic function of the left hemisphere [68,69], (2) the aprosodias are a universal phenomenon and (3) the ability to manipulate F_0_ over time to produce affective prosody in English may not be acoustically valid for speakers of tone languages.

Using the above-described computer program for acoustically analyzing affective prosody (see last paragraph of Section 2.3), my colleagues and I published a series of papers assessing affective–prosodic production in speakers of tone and non-tone languages, including subjects with RBD [62,66,70,71]. The first study was published in 1986 by Ross, Edmondson and Seibert [66]. Twenty neurologically intact individuals whose native language was either English, Mandarin, Thai, or Taiwanese were evaluated on the affective repetition task developed previously [60] that was translated into the appropriate language for testing. The most powerful finding was that English speakers manipulated F_0_ to a far greater extent, as measured by F_0_ slope, variation and delta, for signaling affective prosody compared with the tone language speakers [main effect *p*-values < 0.0001 and post-hoc tests showing English > (Mandarin, Taiwanese, Thai) with *r*^2^ effect sizes all greater than 0.60 as calculated from the published means and SDs]. Interestingly, no statistical differences among subject groups were found for F_0_ register. Thus, it was concluded that tone language speakers compared with English (non-tone) speakers have limited ability to manipulate F_0_ because doing so would alter lexical tones and tonal relationships which, in turn, would cause aphasic-like phonological and semantic disruptions. However, tone language speakers are able to increase or decrease their overall mean pitch (F_0_ Register) to signal affect because doing so does not alter tonal relationships.

The next study, published in 1988 by Ross, Edmondson, Seibert and Homan [62], assessed English-speaking patients undergoing a Wada test [72] in preparation for potential neurosurgery to remove epileptic tissue. The study was made possible because Richard Homan (Assist. Prof. of Neurology at University of Texas Southwestern Medical School) had developed a regional Epilepsy Center at the Dallas VA Hospital and we had decided to engage in collaborative research. The Wada test involves hand-injecting sodium amytal into the right and left carotid artery to reversibly anesthetize regions of the ipsilateral hemisphere that are irrigated by the anterior and middle cerebral arteries to determine how propositional language and memory are lateralized in the brain. Five right-handed patients were evaluated on spontaneous affective prosody and their ability to repeat the sentence “I am going to the movies” using neutral, sad, happy, surprised, bored and angry intonations by imitating the examiners voice. During the right-side Wada test, all patients became affectively flat. During the left-sided Wada test, all patients had the onset of severe non-fluent aphasia and their affective–prosodic repetition could not be formally tested. Their performance on the repetition task underwent acoustic analysis. For each patient, the mean and standard deviations across the six affects were calculated for each acoustic measure for each experimental condition. The data then underwent a repeated one-way ANOVA with the thirteen acoustic measures serving as the dependent variable and the experimental condition (before, during and after the Wada test) serving as the independent variable. Based on post-hoc analyses, all acoustic measures after the Wada test were statistically indistinguishable when compared with the pre-Wada condition. The only measures that statistically differentiated pre-Wada from Wada performance in decreasing robustness, calculated from the published means and SDs, were F_0_ delta (*r*^2^ = 0.73), F_0_ register (*r*^2^ = 0.72), F_0_ slope (*r*^2^ = 0.63) and F_0_ variation (*r*^2^ = 0.56). Thus, the research established that in English, a non-tone language, the ability to modulated pitch over time was the most salient acoustic signal underlying affective prosody.

The third study in the series was published by Edmonson, Chan, Seibert and Ross [70] in 1987 and involved eight right-handed Taiwanese patients with acute ischemic infarctions involving, at minimum, the right fronto-parietal operculum based on CT scan that resulted in either motor or global aprosodia and eight neurologically intact controls. This research was possible because, at the time, Jin-Lieh Chan (Neurologist, Department of Neurology, Chang Gung Memorial Hospital, Taipei, Taiwan) was completing a Behavioral Neurology Fellowship with me at Southwestern Medical School in Dallas, TX. He is the same Chan who was second author in the Hughes, Chan and Su publication [67] that first described aprosodias in Mandarin-speaking patients after focal RBD. We were able to have Jin-Lieh return to Taiwan to collect, test and tape record the subjects’ responses using a standardized affective repetition task that he tape recorded in Taiwanese. The recorded task was the verbal equivalent of “You are going to the movies” spoken with either a neutral, angry, sad, surprised, or happy affect. None of the patients had aphasia or disruption of lexical tones by formal examination and all had markedly flat affect. Six acoustical measures were found to statistically differentiate patients from controls in decreasing robustness (calculated from the published *t*-test values and degrees of freedom): F_0_ register (*r*^2^ = 0.86), F_0_ attack (*r*^2^ = 0.68), F_0_ delta (*r*^2^ = 0.63), dB register (*r*^2^ = 0.59), Total time (*r*^2^ = 0.53) and dB delta (*r*^2^ = 0.41) that were clearly different from the within subjects Wada study in English-speaking patients [63]: F_0_ delta (*r*^2^ = 0.73), F_0_ register (*r*^2^ = 0.72), F_0_ slope (*r*^2^ = 0.63) and F_0_ variation (*r*^2^ = 0.56). We had anticipated that F_0_ register, total time and, some dB-related measures (register, delta) would acoustically characterize the loss of affective prosody after RBD based on our previous research [62] and when listening to the tape recordings that had undergone acoustical analysis. The changes in F_0_ attack and F_0_ delta were unexpected. However, these results can be easily accounted for because Taiwanese speakers use Total time to convey affect in their speech. If the same sentence is spoken quickly for affective purposes, then any rises or falls associated with lexical tones will occur more rapidly and alter the metrics of F_0_ attack and also F_0_ delta, as it is derived based on a statistical comparison with the neutral repetition that is spoken at a natural pace. Thus, in Taiwanese, the most salient acoustical means of conveying affect is by altering overall pitch, varying loudness and the speed of articulation.

The final paper in the series was published in 1992 by Ross, Edmonson, Seibert and Chan [71] to address the issue of “tone latitude” and its possible acoustic contribution to affective prosody. Tones are considered to be perceived categorically. From a psychoacoustic perspective, what this means is that minor variations in intonation associated with lexical tones will not alter the linguistic perception of that tone. For example, if a high flat tone rises slightly or has a slight rise and fall or is slightly higher than expected in its relationship to other tones, it is still understood semantically as a high tone. To explore if tone latitude is used in Taiwanese for affective–prosodic signaling, the tape-recorded utterances that were gathered in the previous study [70] were segmented into six syllables, and four measures of lexical tone were performed for each word: average F_0_, initial F_0_, F_0_ slope and length F_0_ (the amount of time the syllable was voiced). An emotional range was calculated for each syllable across each affective condition for each subject that underwent a two-way repeated-measures ANOVA. The statistical results were somewhat complex but demonstrated that tone latitude was diminished in the RBD patients. Overall, the emotional range of average F_0_ was reduced by 44%, initial F_0_ by 38% and F_0_ slope by 46% in the RBD group compared with controls. The *r*^2^ effect sizes, calculated from the means and standard error of the means, were quite robust, ranging from 0.55 to 0.67 [42], thus lending support to the hypothesis that tone language speakers may use tone latitude as a means to acoustically signal affective intent in their speech. A psychoacoustic perceptual study, however, needs to be carried out to fully validate this hypothesis.

In summary, different acoustical features underly production of affective prosody in tone versus non-tone languages. This is an important finding because a number of publications, based on data obtained from English-speaking subjects, have theorized that hemispheric lateralization of language functions are driven by each hemisphere having innate (hard-wired) abilities to differentially process acoustical information, with the left hemisphere being better at short acoustic signals associated with consonants and vowels and the right hemisphere being better at long acoustic signals associated with intonation [73,74,75,76]. Our studies in tone and non-tone languages, however, do not support these theories. In addition, the publications by Dehaene-Lambertz and colleagues [77,78], using high-density event-related potentials, did not find differential hemispheric processing of short speech sounds (consonants and vowels) versus long intonations in neonates prior to their acquisition of language. Therefore, one must inductively conclude that the “… communicative abilities of humans are lateralized according to the behavior itself (affective vs. verbal–linguistic) and not according to the physical/acoustical carrier that expresses this behavior … Thus, human languages show the features of a composite that is the product both of specific neurological organization of brain tissue and of the brain’s ability to react to the acoustical properties of a particular language, i.e., tone vs. non-tone, during the experience of language acquisition” (p. 232; Edmondson, Chan, Seibert and Ross, 1987) [70].

### 2.5. Is Affective Prosody a Dominant and Lateralized Right-Hemisphere Function?

Starting in the mid-1970s, a number of investigators have published studies describing deficits in the modulation of affective prosody after LBD. Schlanger, Schlanger and Gerstmann [79] assessed 20 patients with RBD and 40 patients with LBD who had either severe or mild aphasic comprehension deficits. They reported that RBD and LBD patients with severe comprehension deficits were statistically more impaired than LBD patients with mild comprehension deficits when asked to identify the emotion conveyed by a neutral or semantically meaningless sentence presented with either a happy, sad, or angry emotion. The only difference in performance that was statistically different was between LBD patients with mild versus severe comprehension. However, the actual differences among groups were quite small: LBD patients with mild comprehension deficits responded correctly 77% of the time, RBD patients responded correctly 70% of the time and LBD patients with severe comprehension deficits responded correctly 67% of the time. It was reported that normal controls respond correctly 97% of the time to the task. In 1982, Seron, Van der Kaa, Vanderlinden, Remits and Feyereisen [80] tested the ability of 27 patients with either Wernicke, global, or Broca aphasia on their ability to identify the emotion conveyed by a neutral or a semantically meaningless sentence presented with either a happy, sad, or angry emotion or in sentences in which the affective prosody was either consistent or inconsistent with the verbal emotional content. They found that the ability of aphasics to comprehend affective prosody compared with normal controls was impaired on the various emotional tasks and that their performance was related to the severity of the aphasic comprehension deficit. In 1985, De Bleser and Poeck [81] observed that emotional intonation was restricted in patients with severe global aphasia whose verbalizations were limited to recurrent consonant–vowel syllables in contradiction to the observations of Jackson [35,36,37]. In 1990, Cancelliere and Kertesz [82] reported that various aprosodic syndromes may occur after LBD and noted that some of the patients had only mild aphasic deficits.

Two formal clinical assessments have been developed to quantitate affective communication and determine if affective prosody is a lateralized function of the right hemisphere. The Florida Affective Battery (FAB) was published in 1989 by Bowers, Blonder and Heilman [83]. It uses various facial expressive, vocal and cross-modal stimuli to assess subjects on a variety of tasks that include discrimination, naming and matching. The FAB can be administered to most brain-damaged patients as long as they do not have aphasic deficits that are severe enough to interfere with formal testing [32,84]. The Aprosodia Battery (ApB) was developed in 1997 by Ross, Thompson and Yenkosky [32] to specifically address hemispheric lateralization of affective–prosodic functions. Unlike the FAB, the ApB does not formally assess comprehension or production of facial expressions but can be administered to most patients with aphasia, regardless of severity.

Using the FAB, Bowers and colleagues [55] published a deductive research study to determine if loss of emotional prosodic comprehension after RBD was due to a fundamental processing deficit versus a distraction deficit related to the verbal semantic elements that were incorporated into their test stimuli. The research involved right-handed patients with focal RBD and LBD caused by ischemic infarctions. The first experiment assessed patients and controls using three test conditions: (1) sentential stimuli in which the verbal semantic emotional information matched the affective–prosodic emotional signal, (2) the affective–prosodic emotional signal was inconsistent with the emotionally neutral verbal–semantic information and (3) the affective–prosodic emotional signal was in conflict with the verbal semantic emotional information. The second experiment tested subjects on semantically neutral sentences with different affective–prosodic emotions and the same sentences that were acoustically filtered so that frequencies less than 100 Hz and greater than 5000 Hz were removed to make it more difficult to comprehend the verbal–linguistic but not the emotional prosodic content. Although the statistical analysis of results was quite complex [42], the major conclusion was that the emotional comprehension deficits in RBD patients were a combination of a processing and a distraction effect. In contrast, the emotional comprehension deficits in LBD patients were largely due to a distraction effect related to the presence of aphasia.

In a follow-up study, Blonder, Bowers and Heilman [85] assessed right-handed patients with focal RBD and LBD caused by ischemic infarctions using various subtests of the FAB. The most crucial experiment involved the ability to infer the emotional meaning of a sentence from its verbal–linguistic content spoken with neutral affective prosody or to infer the appropriate facial (emotional) expression based on the verbal–linguistic content of a sentence spoken with neutral affective prosody. They found that RBD patients who had deficits in comprehending emotional prosody and facial expressions were able to infer the appropriate emotion and facial expression when presented with sentences communicating emotions using only verbal–linguistic descriptions. Based on their findings and those of the previous study [55], they concluded that loss of the ability to comprehend emotional prosody (and facial expressions) in patients with RBD was caused by destruction of “nonverbal communicative representations”, i.e., a primary processing deficit similar to the concept that aphasias following LBD are caused by destruction of verbal–linguistic representations [15,85].

The ApB [1,32] was developed based on the following (inductive) reasoning and the assumption that the verbal–linguistic aspects of language are mediated primarily by the left hemisphere and the affective–prosodic aspects of language are mediated primarily by the right hemisphere. If this assumption is correct, then, prior to any speech act, the hemispheres must be able to condition each other to produce speech whose verbal–articulatory and affective–prosodic components are temporally and behaviorally coherent. For example, if a speaker wants to produce a happy or boastful statement, then the right hemisphere needs to be appraised by the left hemisphere of the word order and tempo of articulation so that it can temporally match the intended affective–prosodic signal with the articulatory line. Conversely, if the right hemisphere intends to stress a certain word or phrase for conveying attitudinal or emotional intent, then the left hemisphere will need to be appraised by the right hemisphere so that the articulatory timing will correctly match the intended stress or intonation pattern. This preconditioning occurs before the respective hemispheres generate their motor output which are then integrated in the brainstem motor nuclei that control muscles used for speech production [86,87]. Therefore, deficits in modulating affective prosody associated with LBD could be the result of three possibilities: (1) aphasic non-fluency that impairs the timing and verbal–articulatory aspects of speech, (2) aphasic comprehension deficits that interfere with formal testing of affective prosody and (3) lesions that disrupt interhemispheric interactions that are coordinated through the corpus callosum. In order to assess these three possibilities, the ApB uses stimuli with incrementally reduced verbal–articulatory demands to test affective–prosodic repetition and comprehension. The word subtests use the sentence, “I am going to the other movies”, with lexical stress on either the “am” or “other” to carry neutral, happy, sad, indifferent, angry and surprised affects. The monosyllabic subtest uses a repeated syllable, “ba..ba..ba..ba..ba..ba..ba”, with lexical stress on either the second “ba” or the fifth “ba” to carry the six aforementioned affects. The asyllabic subtest uses a vocalic, “aaaaaaaaaahhhhhh”, with lexical stress either early or late to carry the aforementioned affects. When assessing comprehension, there is also a discrimination subtest, in which the subjects are asked if paired word stimuli, one with lexical stress early and one with lexical stress late, have the same or different affect. The reasons for using stimuli with incrementally reduced verbal–articulatory demands are twofold: to shift the task from being bi-hemispheric to a predominantly right hemispheric, which was subsequently confirmed by a functional MRI study [88], and to minimize any aphasic deficits that could interfere with the formal assessment of affective prosody. There is also an assessment of spontaneous affective prosody in which the subject is asked to recall an emotional life event that is tape recorded. Ten seconds were analyzed acoustically using the PM Pitch Analyzer connected to a personal computer. (Note: the PM Pitch Analyzer is no longer manufactured, but the acoustic analyses can be accomplished by using digital software, such as Pratt [89], and importing the acoustic data into an Excel spreadsheet (Microsoft, Inc., Redmond, WA, USA) for analysis [50]). The repetition tasks are also analyzed acoustically by extracting the F_0_ in Hz across each utterance and quantitating pitch variability by calculating the percent F_0_ coefficient of variation [(Hz mean/Hz SD) × 100; F_0_-CV%]. Subsequent research has shown that acoustic measures of F_0_ variability, such as F_0_-CV%, are strongly correlated to subjective ratings of affective–prosodic intensity [90].

**Figure 2:**
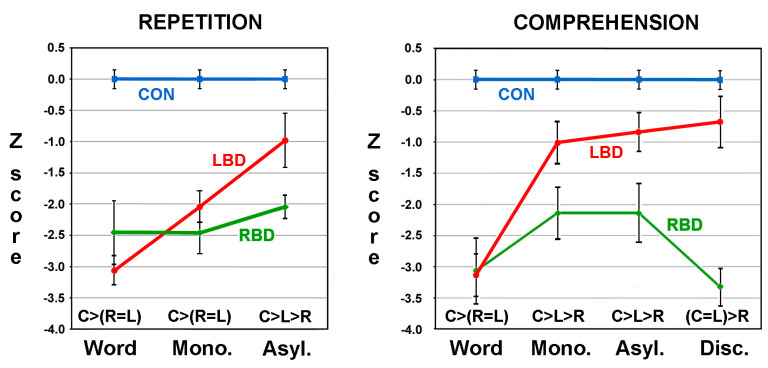
Graphed results comparing the repetition and comprehension performance of controls (CON, C) and impaired RBD (R)) and LBD (L) patients with Z-scores of <−1.64 on the word tasks (Mono. = monosyllabic, Asly. = Asyllabic, Disc. = Discrimination). The post-hoc statistical relationships are shown just above the abscissa. Variance hats represent SEMs. Adapted from means and SEMs published in reference [33].

Two papers were published using the ApB. The first was a proof of concept that established the utility of the ApB in differentiating aprosodic deficits in RBD and LBD patients [32]. The second paper by Ross and Monnot in 2008 [33], using a larger number of subjects, confirmed and extended the initial findings reported in the first paper. Eighteen right-handed patients with LBD and twenty-one right-handed patients with RBD due to ischemic infarctions were evaluated on the ApB and the Westen Aphasia Battery (WAB) [20] between three- and eight-weeks post-stroke. All patients underwent MRI scans 2–6 weeks post-stroke. The F_0_-CV% acoustic measures obtained on spontaneous task and the various repetition subtests and the raw scores obtained on the various comprehension subtests were converted to Z-scores based on the performance of 43 age-equivalent controls [(subject’s F_0_-CV% or score—control mean)/control SD]. The patient group’s results were stratified into those with affective–prosodic deficits (Z-score of <1.64) and those without an aprosodic deficit (Z-score of ≥1.64). On spontaneous affective prosody, eight LBD patients (44%) and twelve RBD patients (57%) were impaired on the task with both groups having a Z-score mean of approximately −3.0 that was not statistical different from each other. The repetition tasks underwent a repeated-measures ANOVA with the research groups serving as the independent variable and the Z-scores for the word, asyllabic and monosyllabic tasks serving as the dependent variable. Thirteen RBD patient (62%) and nine LBD patients (50%) had a Z-score of <−1.64 on the word task and were stratified into the impaired patient groups. A significant main effect for groups was observed that was very robust with a partial *η*^2^ effect size of 0.60, a significant main effect for the task (partial *η*^2^ effect size of 0.12) and a significant task by group interaction (partial *η*^2^ of 0.23). Post-hoc analyses demonstrated that the performance of RBD patients did not improve across the tasks whereas the performance of the LBD patients improved to near normal (Figure 2). The comprehension tasks also underwent a repeated-measures ANOVA. Sixteen RBD patients (76%) and ten LBD patients (56%) had a Z-score of <−1.64 on the word task and were stratified into the impaired patient groups. There was a significant main effect for groups that was very robust with a partial *η*^2^ of 0.60, a significant main effect for the task (partial *η*^2^ of 0.12) and a significant task by group interaction (partial *η*^2^ of 0.19). Post-hoc analyses showed that the performance of RBD patients did not materially improve across the tasks whereas the performance of the LBD patients improved to normal (Figure 2).

Further analysis of the data showed that there were no statistically significant relationships between aprosodic deficits and aphasic deficits, consistent with prior publications [32,91], in LBD patients who were impaired in either the spontaneous, word repetition, or word comprehension tasks of the ApB. In LBD patients, the only lesion location statistically associated with impaired affective–prosodic production involved the white matter situated just below the supplementary motor area and adjacent to the anterior body of the corpus callosum. No lesion locations were found to be statistically associated with impaired affective–prosodic comprehension. In contrast, lesion locations were highly predictive of affective–prosodic deficits in RBD patients. Impaired spontaneous (affective prosody) and repetition were highly correlated with lesions involving the posterior frontal operculum (analog of Broca’s area). Impaired comprehension was highly correlated with lesions involving the posterior temporal operculum (analog of Wernicke’s area). The only unexpected finding was that impaired affective–prosodic repetition was not highly correlated with lesions injuring the right posterior temporal operculum, whereas impaired verbal repetition is highly correlated to lesions injuring the left posterior temporal [92]. The reason for this difference may relate to faster recovery of affective–prosodic repetition after lesions injuring the posterior temporal operculum because of hemispheric differences in the anatomy of the arcuate fasciculus. The arcuate fasciculus is thicker in the left hemisphere and has two distinct fiber bundles: one that directly connects Wernicke’s and Broca’s area and one that indirectly connects Wernicke’s and Broca’s area via synaptic connections in the inferior parietal lobe [30,93]. In contrast, the right arcuate fasciculus is composed, almost exclusively, of indirect connections between the posterior temporal operculum and the frontal operculum that synapse in the inferior parietal lobe [30], perhaps allowing for rapid recovery of affective–prosodic repetition after injury to the temporal operculum, resulting in transcortical sensory rather than a sensory aprosodia [33]. Lastly, we did not find that loss of comprehension was associated with left-sided neglect even though this relationship had been reported previously [94].

Based on the results outlined above, it was concluded that aprosodic deficits following LBD are not due to aphasic deficits per se or lesion locations that typically cause aphasic deficits. However, white matter lesions adjacent to the anterior corpus callosum are associated with impaired affective–prosodic production in LBD patients which substantiates the hypothesis that affective–prosodic deficits may be caused by loss of interhemispheric interactions prior to the speech act. Finally, reducing the verbal-articulatory demands on LBD patients improves their performance on (affective–prosodic) repetition and comprehension to normal or near normal in contrast to RBD patients, thus strongly supporting the following hypotheses: (1) affective prosody is a dominant and lateralized function of the right hemisphere, (2) affective–prosodic deficits observed in patients with LBD are not true aprosodias and (3) the aprosodias observed in patients with RBD are due to disruption of affective communication representations similar to the concept that the aphasias are due to disruption of verbal–linguistic representations in the left hemisphere [55,85].

### 2.6. Are the Aprosodias Functionally and Anatomically Analogous to the Aphasias?

Based on the initial descriptions of patients with affective–prosodic deficits after focal RBD [38,39,40] that ultimately led to the concept of aprosodic syndromes [41], it was posited that the aprosodias were functional–anatomic analogs of the aphasic syndromes. Subsequent publications have, for the most part, confirmed this supposition as long as two provisos are met: (1) the lesions are acute and relatively focal, such as ischemic infarctions and small hemorrhages, as opposed to large lesions, such as intracerebral hemorrhages, that cause brain distortions and extensive edema, or slowly growing lesions, such as tumors, that allow time for concomitant functional recovery to occur, and (2) the functional–anatomic correlations are carried out after acute effects, such as diaschisis, edema and ischemic penumbra, resolve (approximately 2 weeks post injury) and before the onset of long-term recover (approximately 6 weeks post injury) [15,20,33,95,96,97]. In addition, MRI scans are preferrable to CT scans for functional–anatomic correlations because they more accurately define lesion extent by detecting small lesions that may involve the opercular cortex and areas of ischemic damage that are not overtly infarcted and also by avoiding the “fogging effect” on CT scans in which hypodense infarcted tissue may become iso-dense approximately two to three weeks post-stroke before becoming hypodense at a later date [33,98,99,100]. The fogging effect may also occur on T2-weighted MRI sequences but not on other sequences, such as FLAIR or DWI [101]. Motor aprosodia, analogous to Broca aphasia, is most commonly due to acute lesions that involve, at minimum, the right frontal operculum, with or without injury to the basal ganglia [15,33,40,41,44,82], but may also result from lesions limited to the basal ganglia [82,102,103]. Sensory aprosodia, analogous to Wernicke aphasia, is most commonly due to acute lesions that involve, at minimum, the posterior temporal operculum [15,33,86,104,105,106] but may also result from lesions limited to the thalamus [107]. Transcortical motor aprosodia, analogous to transcortical motor aphasia, is most commonly due to acute lesions that involve the superior medial frontal region that is irrigated by the anterior cerebral artery [108,109,110]. The functional–anatomic correlates of transcortical sensory aprosodia are complex and related to widely distributed cortical lesions that may also include deep structures [41,44,82] and have also been reported after lesions involving the thalamus and the posterior temporal operculum that one would have expected to result in sensory aprosodia [33]. The publications that have not supported the above functional–anatomic relationships have either examined patients many months after brain damage or used patients with various types of brain tumors or large intracerebral hematomas [33].

However, there is a lesion localization that has been associated with aprosodic but not aphasic deficits. Bilateral or unilateral injury to the orbito-frontal and/or adjacent medial frontal regions of the brain may cause impairments in comprehending emotional prosody using test stimuli, in which the affect is carried by articulated voiced speech sounds that are devoid of consonants [111,112]. The ability to imitate (repeat) the test stimuli is preserved, suggesting a transcortical type of deficit [33]. The mechanism underlying the loss of affective–prosodic comprehension after medial orbitofrontal lesions has not been assessed using either the FAB or the ApB to determine if it is a primary processing deficit, attributable to disruption of affective representations [33,55,85], a secondary language processing deficit similar to that observed after LBD [33], or related to attentional or cognitive impairments associated with orbito-frontal lesions that may interfere with formal testing, such as hasty decision making, poor insight, confabulation, or impaired error detection [113].

It should also be noted that two functional imagining studies have localized affective–prosodic comprehension to the right anterior frontal operculum [114,115]. The reason for this erroneous localization is the use of high-level subtraction techniques [33,42]. All functional imaging studies that have examined the processing of language in the brain using either affectively neutral or affectively driven, verbal–linguistic, stimuli [88,116,117,118] have observed bilateral, relatively symmetric, activations of the posterior temporo-parietal opercular regions using low-level subtractions (stimulated state minus resting state), even though lesions of the left posterior operculum are not associated with aprosodic comprehension deficits and lesions of the right posterior operculum are not associated with aphasic comprehension deficits [19,20,21,33]. In a complex fMRI study exploring verbal and emotional-prosodic discrimination, Buchanan and colleagues [115] observed the requisite bilateral posterior opercular activations on low-level subtraction and also bilateral pre-frontal opercula activations thought to be related to task demands and working memory. In order to get rid of the bilateral activations, the subjects were re-scanned using a purely verbal task and a purely emotional task. When the emotional task was subtracted from the verbal task (high-level subtraction), verbal comprehension was now associated with activation of the left pre-frontal opercula region. When the verbal task was subtracted from the emotional task, emotional comprehension was now associated with activation of the right pre-frontal opercula region. Although focal brain damage involving the frontal operculum has never been implicated in the overt comprehension of either the verbal or emotional aspects of language [15,19,20,21,33], it was concluded that the right frontal operculum was essential for emotional prosodic comprehension and the left frontal operculum was essential for verbal comprehension. In a similar study using PET scanning and high-level subtraction techniques, George and colleagues [114] also localized comprehension of affective prosody to the right frontal operculum. Thus, one has to be very skeptical of using functional imaging as a means to localize brain functions, especially if the localizations are not consistent with traditional functional–anatomic relationships [15,33,42].

Although there are some minor differences, specifically that affective–prosodic repetition is not highly correlated to lesions involving the right posterior temporal operculum [33], or unresolved issues, specifically the mechanism underlying impairment of emotional prosodic comprehension after medial orbito-frontal injury [33,113], it is reasonable to conclude that from a functional–anatomic perspective, the aprosodic syndromes associated with focal RBD are analogous to the aphasic syndromes associated with focal LBD (see Figure 1, right panel).

### 2.7. Neurology of Linguistic Prosody

There has not been a great deal of research on the neurology of linguistic prosody. The publications by Monrad-Krohn [47,48] that focused on the prosodic aspects of language and communication were initiated by a patient he encountered in the clinic. The patient had suffered a shrapnel injury to her left skull during WWII that resulted in Broca aphasia with severe non-fluency. The patient’s non-fluency rapidly recovered but she was left with a “foreign” accent. By birth, the patient was a native Norwegian. However, her newly acquired accent was perceived by other Norwegians as Germanic in origin, causing her to be socially ostracized because, at that time, Norway was occupied by Nazi Germany. Monrad-Krohn observed that her overall melody of speech, including the ability to sing, emote and intone affect, was preserved. In contrast, her speech was characterized as having inappropriate pronunciation, timing and stressing of words. Subsequent research has established that the foreign accent syndrome is the result of LBD, most commonly involving the frontal operculum and/or basal ganglia, in which aphasic non-fluency does not completely recover [119]. Other research, however, has reported that brain damage to either hemisphere may lead to disturbances in the modulation of linguistic prosody [50]. A number of publications have described deficits in the production of emphatic (lexical and/or contrastive) stress after focal brain damage. For example, Weintraub, Mesulam and Kramer [120] reported that RBD patients were significantly impaired in their production of emphatic stress and also sentential intonation (terminal rise in pitch) that differentiates a declarative from an interrogative sentence. Ouellette and Baum [121] found that controls, LBD patients with non-fluent aphasia and RBD patients were able to produce lexical and contrastive stress using appropriate modulation of pitch and loudness but the LBD patients had difficulty modulating appropriate durational changes. Unfortunately, the stress production was not rated for intensity.

In order to more completely address the neurologic basis for linguistic stress, Ross, Shayya and Rousseau [50] published a study that acoustically analyzed lexical stress production in 18 right-handed LBD patients, 20 right-handed RBD patients and 40 age-equivalent controls who had been evaluated previously for their ability to modulate affective prosody [33]. When testing affective–prosodic repetition, the ApB uses affectively driven stimuli that also have lexical stress placed either early or late in the “sentence”. The affectively neutral monosyllabic (“ba..ba..**ba**..ba..ba..ba..ba”) repetitions were analyzed rather than the affectively neutral word repetitions because it was much easier, more precise and more reliable to acoustically define and measure lexical stress in the monosyllabic repetitions [122]. The stressed “ba” syllable and the “ba” syllables before and after were defined by programmable cursors using Pratt software [89] and their F_0_ and dB contours and timing data were exported to an Excel spread sheet (Microsoft, Inc.). Eight acoustic measures were derived to characterize each syllable: semitone mean, SD and slope, decibel mean, SD and slope, (syllabic) time and (pre-syllable) pause time. Two delta metrics were calculated for each acoustical measure: D1 (stressed minus pre-stressed syllable) and D2 (post-stressed minus stressed syllable). This was performed to remove variability due to individual differences in natural F_0_, dB and articulatory speed amongst subjects and differences in overall dB due to microphone placement and tape-recording level. In addition, the intensity (stress prominence) of the stressed syllable was rated for saliency by 10 judges, using a five-point Likert scale. The important findings relative to the neurology of lexical stress are as follows. Lexical stress can be impaired by either RBD or LBD, but the prevalence is small with only 28% of LBD and 35% RBD patients being judged as impaired. This suggests that lexical stress is not lateralized in the brain and that it is a highly distributed brain function, making it fairly resistant to focal RBD and LBD. The only lesion location found on the MRI scan that was statistically associated with impaired stress prominence involved the left basal ganglia. Based on a series of stepwise discriminant analyses, loss of stress prominence, as expected, was very robustly associated with aphasic non-fluency in LBD patients (*V*^2^ effect size of 0.95), as quantitated by the WAB. In contrast, loss of stress prominence was not associated with deficits in affective–prosodic repetition, as measured by the ApB, in either patient group. However, the presence of affective–prosodic repetition deficits differentially condition the acoustic signature of lexical stress. In LBD patients, stress prominence was positively and highly correlated with decibel SD D1 (R^2^-adjusted effect size of 0.80). In RBD patients, stress prominence was positively and highly correlated with semitone mean D1 (R^2^-adjusted effect size of 0.93), a very unexpected finding, since the patients had lost the ability to vary pitch over time for affective–prosodic purposes. Thus, the above findings strongly suggest that the neurology of linguistic prosody, specifically lexical stress, and affective prosody are fundamentally different.

### 2.8. Other Right-Hemisphere Contributions to Language and Communication

Although the concept that affective prosody is a lateralized function of the right hemisphere, preliminary research has suggested that the right hemisphere may also contribute to other aspects of language. These include its ability to comprehend the connotative (non-standard) as opposed to the denotative (standard) meaning of words, to understand thematic (overarching) intent conveyed during discourse or written paragraphs that supersedes sentential information and to process complex linguistic relationships, idiomatic or nonliteral types of expressions, metaphor and cursing [35,36,37,123,124,125,126,127,128,129,130,131,132,133,134,135,136]. These observations also lend strong support to the concept that human language is a truly bi-hemispheric brain function [15].

## 3. Neurology of Depression and Hemispheric Lateralization of Emotions

### 3.1. Academic and Clinical Serendipity

In the late 1970s, when I was an Assist. Prof. of Neurology at the University of Texas Southwestern School of Medicine in Dallas, I met John Rush who was an Assoc. Prof. of Psychiatry because of our common research interests. His research was focused on the biological underpinnings of affect disorders, specifically depression and manic-depressive illnesses, whereas my research was focused on the neurology of affect and emotions. For a number of years, we educated each other about our respective disciplines and discussed the possibility of collaborative research. John and his colleagues were instrumental in developing and validating that a regimented form of verbal cognitive (talk) therapy is an effective method for treating patients with non-endogenous (exogenous, non-melancholic) types of depression that is superior to pharmacological interventions [137,138,139]. He also educated me about current psychiatric research that was using the dexamethasone suppression test (DST) [140,141,142,143,144,145] as a potential biological marker for endogenous (melancholic) depression. Patients with endogenous depression [146,147,148,149,150] are more likely to be hospitalized than patients with non-melancholic depression. The patients are characterized by having an overt depressive affect with psychomotor retardation that generates empathy because they appear to be clinically ill. The depression is often accompanied by vegetative symptoms, such as anorexia, weight loss and insomnia, suicidal ideation and pervasive anhedonia. The patients usually have no ready verbal explanation for why they are feeling so depressed, suggesting that the depression is triggered internally by genetic or biochemical factors rather than external events, a distinction that may not be valid (see below, last paragraph of this section). In general, patients with endogenous depression do not respond to cognitive therapy but will respond to tricyclic antidepressants, monoamine oxidase inhibitors or electroshock therapy. In contrast, patients with exogenous (non-melancholic) depression [146,148] are more often managed in the out-patient setting. They usually do not evince an overtly depressive affect or have significant vegetative symptoms but may have suicidal ideation. However, they have extensive verbal–cognitive explanations as to why they feel depressed that are usually attributed to external psychosocial problems related to work, marriage, finances, social status or underlying neuroses (Note: the classification of depression into endogenous and exogenous categories has gradually fallen out of favor after the nosology was dropped in the third edition of the Diagnostic and Statistical Manual (DSM-III) [151] and replaced by Major Depressive Disorder; however, the concept of endogenous and exogenous still has clinical validity to help direct appropriate treatments and arguments have been made that the distinction should be re-instated in future revisions of the DSM [146,148,149,152,153]). From a behavioral neurology perspective, I immediately speculated that endogenous depressions are primarily a right-hemisphere disorder, whereas exogenous depressions are primarily a left-hemisphere disorder. However, at that time, there were two major hypotheses regarding emotions and their hemispheric lateralization [4,5]. The right-hemisphere hypothesis postulated that emotions and related display behaviors are a lateralized function of the right hemisphere and the valance hypothesis postulated that negative emotions and related display behaviors are a lateralized function of the right hemisphere and positive emotions and related display behaviors are a lateralized function of the left hemisphere. Although these two hypotheses are supported by extensive clinical research, they are mutually exclusive and did not lend support to the idea that exogenous depression, a negative emotional state, could be a left-hemisphere disorder. Unfortunately, my colleagues and I [5] had not yet formulated, based on unexpected observations during our right-sided Wada study (see Section 2.4) [62], the emotion-type hypothesis that would have reinforced the idea that exogenous depression was a left-hemisphere disorder. The emotion-type hypothesis postulates that primary emotions and related displays are a right-hemisphere function and social emotions and related displays are a left-hemisphere function (see Section 3.2 below for further discussion regarding social and primary emotions). Nevertheless, the opportunity to engage in collaborative research finally occurred because of an elderly patient who was admitted to the neurology ward at Parkland Memorial Hospital in Dallas, TX, that ultimately led to a 1981 publication titled “Diagnosis and neuroanatomical correlates of depression in brain-damaged patients” [154].

Six weeks prior to her admission to Parkland Memorial Hospital, an 81-year-old woman suffered an ischemic infarction involving the posterior temporo-parietal operculum region that resulted in Wernicke aphasia. She was subsequently placed in a nursing home by her family because they were unable to manage her at home. She was dismissed by the nursing home because of severe behavioral problems that they were unable to manage. This led to a second nursing home placement that also resulted in a dismissal and precipitated her admission to the neurology ward at Parkland Memorial Hospital. She was noted by the nurses to be extremely irritable and negative with explosive behavioral outbursts that occasionally resulted in biting and scratching. She was uncooperative with personal hygiene, refused food, avoided all social interactions and had poor sleep patterns. She was described by the nurses as being a “high holy terror”. After being placed on low doses of haloperidol, according to her daughter, she “quieted down” but was still “seething underneath”. When I evaluated the patient, she just stared out the window, did not try to engage me in conversation and was totally uncooperative with a neurologic examination, including a bedside assessment of language. In addition, she did not respond to any mitigating behavioral interactions, such as speaking to her using a quiet, non-threatening, solicitous affect that I had learned to employ for managing acute behavioral changes in Wernicke aphasics that avoided using neuroleptics or other sedatives or placing the patient on a psychiatric ward [155]. Typically, the acute behavioral changes that may occur in patients with Wernicke aphasia resolve over time. Patients with chronic Wernicke aphasia are characterized as being excessively verbal and they will actively seek social interactions, maintain their personal hygiene and do not have vegetative symptoms, such as anorexia or insomnia. To explain the patient’s behavioral changes, I initially thought she could have suffered a second stroke, an underlying dementia or, perhaps, she was depressed. A CT scan of the brain did not show either generalized or focal atrophy or a second stoke and a metabolic work-up was negative for dementia. On questioning family members, I discovered that her husband had died four years previously and that they had noticed a gradual deterioration in her willingness to engaged in social activities and personal hygiene with the onset of anorexia that resulted in a 30-pound weight loss, insomnia, difficulties with concentration and memory and talking negatively about herself, her life and the future, all signs and symptoms consistent with endogenous depression. At that time, the 1968 DSM-II [156] criteria for diagnosing depression (based on consensus) deemed that a patient had to *verbally* admit to feeling depressed or dysphoric or having pervasive anhedonia. Since the patient had been diagnosed as having Wernicke aphasia, she was consequently unable to verbally admit to depression. Thus, I asked John to evaluate the patient and speak with the family. He confirmed that she was suffering from a severe endogenous depression and thought that the DSM-II criteria requiring a verbal acknowledgement of depression was clinically inappropriate (Note: in 1986, DSM-III [151] dropped the verbal criteria as a necessary requirement for diagnosing depression). The patient underwent a dexamethasone suppression test that was non-suppressive with a 4PM cortisol level of 11.6 µg/dL. The patient was started on 10 mg of amitriptyline per day that was gradually increased to 50 mg per day over two weeks with dramatic resolution of her aberrant behaviors. She became socially interactive, had a positive outlook, began sleeping through the night and taking care of her personal hygiene and started to eat again with a 5-pound weight gain. She actively volunteered to help the nurses manage other patients on the ward, despite her persistent Wernicke aphasia, and became their “favorite” patient. Because of her astonishing improvement in behaviors, I was able to convince the family to take care of her at home. At a four-month follow-up, she continued to have an excellent response to low-dose amitriptyline and mild improvement of her Wernicke aphasia. Based on this patient, we decided to collect patients with focal brain damage who were depressed to assess how brain damage may alter or obliterate various signs and symptoms of depression so that we could develop a nascent neurology of depression.

Over the course of approximately a year, we were able to identify four more patients with brain damage who were suffering from a major depression, three of which had focal brain damage due to ischemic infarctions. Case 2, a 34-year-old man, was hospitalized because of an ischemic infarction involving the left inferior fronto-parietal region that caused a dense left hemiplegia with sensory loss with left-sided neglect that resolved over seven days. He was noted to have a markedly flat affect during social interactions. Approximately 17 days post-stroke, he had the onset of intermittent and uncontrollable episodes of crying and complained that he had no control over these episodes and that they were not triggered by any specific environmental event or reflective of an emotional reaction to an environmental event. This aberrant behavior was consistent with the syndrome of pathological affect that may occur after brain injury, most commonly associated with pseudo-bulbar palsy [157]. In addition, speaking in a monotone, affectless, voice unaccompanied by gestures, he verbally complained that he felt “hopeless”, “depressed” and “helpless”. Over the next two weeks, he also began to complain about suicidal thoughts. Despite his verbal reports of depressive symptoms and suicidal ideation, dutifully noted in his chart by the nurses and neurology residents, no one thought he was truly depressed because his verbal complaints describing his internal mood state were not validated by an appropriate depressive affect. On examination, he had motor aprosodia, resulting in a flat affect that made assessing the depth of his depression (internal mood state) difficult. He verbally expressed fears and concerns about his stroke and how it might impair his ability to return to work. He did not have vegetative symptoms and his DST was normally suppressive, suggesting he was suffering from exogenous depression. He was started on 150 mg of imipramine per day because of suicidal ideation. Over a two-week period, all his depressive symptoms and suicidal ideation, by verbal report, resolved but his flat affect was unchanged.

Case 3, a 70-year-old man, was hospitalized because of an embolic stroke due to atrial fibrillation that resulted in an ischemic infarction involving the right mid-superior parietal lobe, resulting in a mild left hemiparesis that rapidly resolved. One week later, he suffered a second ischemic infarction involving the right frontal operculum that resulted in a severe left hemiplegia with some sensory loss and acute delirium with hallucinations that resolved over the next two weeks. On examination, the patient had motor aprosodia with flattening of affect and he was mildly inattentive. He had multiple episodes of pathologic laughing and crying but denied feeling happy or sad during the episodes. Over the next two weeks, he had the onset of marked insomnia, severe anorexia, lack of interest in ward activities and began telling staff that he was “feeling blue”. He became preoccupied with his wife, who he believed was unfaithful to him and had run off with another man, and having suffered a severe right eye injury during WWII. He also began recalling distressing war experiences. After examining the patient on clinical rounds, a woman who was standing in the back of the room pulled me aside and told me that she was his wife and had visited daily during his hospitalization. She then explained that he had two episodes of severe depression in the past with exactly the same delusions. One episode occurred after WWII and the other occurred after he retired. A DST showed non-suppression of cortisol at 4PM with a level of 13.6 µg/dL, consistent with endogenous depression. Unfortunately, we were unable to start pharmacologic treatment because he had a rapid clinical decline due to ventricular tachycardia, cardiac arrest and a third stroke that ultimately caused his death.

Case 4, a 35-year-old woman, had a subarachnoid hemorrhage and underwent neurosurgical clipping of a left internal carotid berry aneurysm. Post-operatively, she suffered a large right-hemisphere ischemic infarction involving the inferior and lateral fronto-parietal regions that extended into the posterior temporal operculum that caused a severe left hemiplegia with sensory loss. She was followed in neurorehabilitation but gradually became very difficult to work with because of irritability, negativity and unwillingness to participate in rehabilitation activities. I was able to examine her approximately six months post-stroke. She had dense right hemiplegia with sensory loss. Her propositional language was intact, but she had mixed transcortical aprosodia, resulting in a markedly flat affect. However, based on her lesion, I suspected that she originally had global aprosodia that partially recovered to mixed transcortical aprosodia. When I asked her specifically about having depressive symptoms, such as anorexia, insomnia, dysphoria, anhedonia and suicidal ideation, she verbally denied all such symptoms. On a three-month follow-up visit, she was accompanied by her mother, who gave a radically different history. Over the last six months, her mother had observed a dramatic change in her social interactions and vegetative behaviors. The patient had always been very supportive and loving towards her children but was now irritable and began to verbally and physically abuse them. Despite the patient reporting that she “loved to eat” and had no problems sleeping, her mother observed that she now “eats like a bird” and had significant insomnia. Her mother also noted that she avoided social interactions and had the onset of uncontrollable laughing or crying in reaction to trivial environmental stimuli. Her DST was non-suppressive with a 4PM cortisol level of 12.6 µg/dL and she had lost 60 pounds over six months. Despite the patient’s verbal denial of depression and depressive symptoms, we diagnosed her as suffering from endogenous-type depression and started her on 125 mg of desipramine at bedtime. Although her irritability, dependency, anorexia, insomnia, pathologic laughing and crying rapidly resolved, she continued to deny that she had been depressed.

Based on these unique cases, it became apparent that diagnosing depression in brain-damaged patients is not straight forward. If a patient has comprehension deficits due to sensory, global or mixed-transcortical aphasia, obtaining history from family members is crucial since they are not able to verbally report symptoms or respond to questions. If a patient has intact comprehension but is non-fluent due to motor or transcortical–motor aphasia, the appropriate history of symptoms can be obtained by using questions that require a yes or no answer rather than asking open-ended questions. If a patient has a flat affect with intact comprehension of affective prosody due to motor or transcortical–motor aprosodia, clinicians should not discount the patient’s verbal complaints of being depressed, experiencing dysphoria or anhedonia, or having suicidal ideation because the complaints are not accompanied by a depressive affect. If a patient has deficits in comprehending affective prosody due to sensory or transcortical–sensory aprosodia, obtaining history from family members is crucial since they may verbally deny melancholic depressive symptoms when taking a history. Understanding these pitfalls is essential for clinicians to diagnose post-stroke depression whose onset usually occurs between 1-6 months post-stroke [158,159].

**Figure 3:**
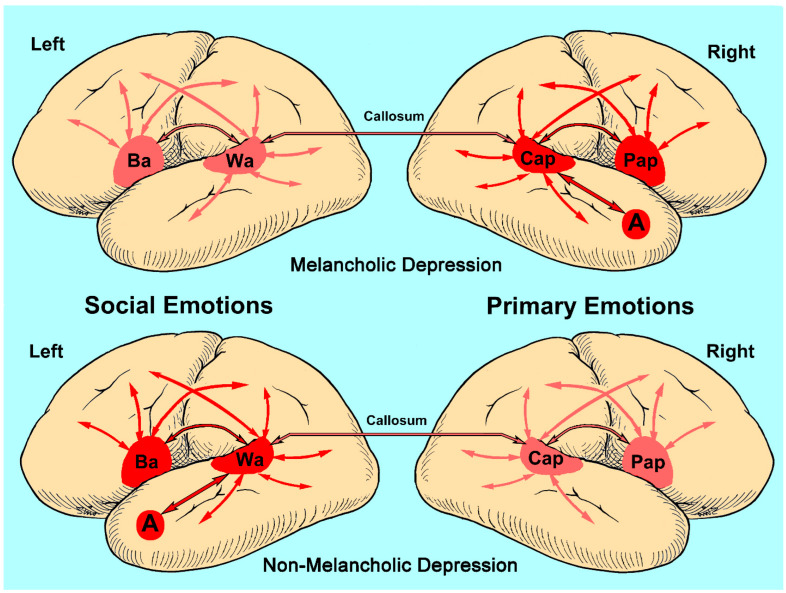
Simplified drawing to illustrate the neurology underlying melancholic and non-melancholic depressions based on the concept that primary emotions and related display behaviors are a lateralized function of the right hemisphere, and social emotions and related display behaviors are a lateralized function of the left hemisphere (A = amygdala, Ba = Broca’s area, Cap = comprehension affective prosody, Pap = production affective prosody, Wa = Wernicke’s area; see text for detailed explanation of the models). The use of bright red indicates the hemisphere that initiates depression and associated display behaviors most likely by the amygdala. The neutral red indicates that the hemisphere is secondarily involved in the depression and the associated display behaviors are less or, perhaps, much less intense than the depressive behaviors generated by the initiating hemisphere. The actual connections amongst areas are complex and not fully illustrated in the diagram (see Figure 1 and Figure 4). For example, each amygdala has extensive parallel distributed connections throughout its own hemisphere, with the densest connections targeting the posterior insula, and also has direct connections to the hypothalamus. In humans and other primates, the temporal limbic structures (amygdala and hippocampal formation) have, at best, meager inter-hemispheric connections (see Figure 4). Also not shown are the extensive intra-hemispheric connections to the pre-frontal and inferior frontal cortices for higher-order cognitive processing of emotions, such as appraisal, that feedback to the temporal limbic region (see Figure 4 and Section 3.2 for details [4]).

We were also able to develop a nascent neurology of depression based on the patients that were diagnosed with endogenous (melancholic) depression (see Figure 3, top panel). The data suggested that the unfolding of endogenous depression is initiated by “a structure or structures essential for … modulating critical features of endogenous depression (e.g., dysphoria, vegetative behavior, hypothalamic dysfunction)” [154]. Current research suggests that the pivotal structure is probably the right amygdala [4]. Once this occurs, via direct and indirect connections to the right posterior temporal operculum, an internal (depressive) affective representation is formed and, via connections to the right frontal operculum, a depressive affect is expressed in both speech and gestural behavior. Vegetative behaviors and endocrine abnormalities, such as a non-suppressive DST, occur via connections between the amygdala and hypothalamus [4]. The left hemisphere becomes involved *secondarily*, via callosal connections between the right and left temporal opercula, and forms a verbal representation of the right hemisphere’s depressive representations that, via connections to the left frontal operculum, allows the verbal–linguistic expression of those representations. If this neurology is correct, it may explain why patients with endogenous depression usually do not have a ready verbal explanation for their depression and why they usually have an intense and empathetic depressive affect. Although we did not offer neurology for exogenous (non-melancholic) depression, based on the emotion-type hypothesis [4,5], one can develop a mirror-image model for non-melancholic depression (Figure 3, bottom panel). Environmental events may trigger a social–emotional depression that is probably initiated by the left amygdala and, via direct and indirect connections to the posterior temporal operculum, forms depressive verbal–linguistic representations. The depressive representation is then expressed verbally via connections to the left frontal operculum. The right hemisphere becomes involved *secondarily* via callosal connections between the left and right temporal operculum and forms a depressive affective representation that is expressed, via connections to the right frontal operculum, during verbal communications through changes in affective prosody and emotional gesturing. If this neurology is correct, it may explain why patients with non-melancholic depression usually have excessive verbal explanations for their depression that are not associated with an intense or empathetic depressive affect. Also implicit in the neurological model is that endogenous (melancholic) depressions could be triggered by environmental events and not just internal genetic or biochemical factors, if those events initiate a primary emotional reaction rather than a social emotional reaction. Thus, the terms melancholic/non-melancholic rather than endogenous/exogenous seem more suitable for classifying depressions. Lastly, based on the neurologic model, patients could exhibit depressions that have mixed melancholic/non-melancholic features, if the environmental events trigger both a social emotional and primary emotional reaction [4] or, as suggested by Rush and colleagues [144], if patients experience recurrent depressions.

### 3.2. Social Emotions, Primary Emotions and the Emotion-Type Hypothesis

In the mid-1980s, when my colleagues and I [62] were assessing the acoustic underpinnings of affective prosody in patients undergoing a right-sided Wada test (see Section 2.4, paragraph 4 for details), we observed unique and unexpected verbal responses. In order to obtain a sample of affectively driven spontaneous speech for acoustical analysis, I would ask patients during their pre-Wada interview to recall a past emotional life event. During and after the right-sided Wada test, the patient was also asked to recall the same life event. Their verbal recall of the factual details of the event before, during and after the Wada test did not change. However, their recall of their emotional reaction to the event during the Wada test was often dramatically different from what they reported pre- and post-Wada test. The very first patient we studied, during his pre-Wada interview, recalled a car accident that caused him to be “… scared, scared to death, I could have run off the road and killed myself or someone else I was really scared”. During the right-sided Wada test, he reported that he felt “silly” and “stupid” about running off the road. When asked directly, he verbally denied being scared or frightened during the event but when asked if he was afraid during the event he replied “… maybe a little” (perhaps, a decathected left-hemisphere memory of the event). The second patient, during his pre-Wada interview, recalled a car accident and being “… scared, I was upset pretty bad about tearing the car … I was very scared mostly when I saw the kids that were playing … that scared me more than anything else because I realized I could have killed them”. During the right-sided Wada test, his initial emotional recall of the event was “I was depressed … about tearing up my car, new car”. When directly asked about other feelings, he finally admitted “I was afraid that I could have killed some little children” (perhaps, a decathected left-hemisphere memory of the event) but denied being scared or very scared during the event. The third patient could not remember a past emotional life event and was not worried about the upcoming Wada test. During the Wada test, when asked about how he felt having the angiogram and Wada test, he replied “… I don’t let anything worry me because if it’s your turn you either live through it or you don’t”, suggesting that he may have been suffering from alexithymia [5]. The fourth patient, during his pre-Wada interview, recalled being “angry” and “frustrated” with past physicians and medical personnel who were evaluating him for possible seizures due to strange auras. During the right-sided Wada test, he recalled feeling “sorry, I felt sorry for people that they had so much trouble finding out what was wrong”. When asked directly, he denied feeling either angry or frustrated. The fifth patient pre-Wada recalled that she had been teased unmercifully by her fellow students and siblings who would call her “stupid” and “dumb” because of her seizures. When asked how she felt about being teased, she immediately and emphatically responded “mad and angry”. During the right-sided Wada, she recalled that her siblings and other children had always “made a lot of fun” about her seizures which made her feel “embarrassed”. When questioned directly, she denied that the teasing caused her to feel angry or mad. After assessing these five patients, (Richard) Homan and I collected six more patients to document changes in emotional recall during the right-sided Wada test that turned out to be very similar. We originally thought that we had uncovered a method for studying repressed memories. However, I intuitively realized our serendipitous observations were somehow fundamental for understanding how emotions are lateralized in the brain since they could not be explained by either the right-hemisphere or valence hypotheses. If the right-hemisphere hypothesis was correct, then anesthetizing the right hemisphere should not have resulted in the recall of a different emotion, such as feeling silly, stupid, depressed, sorry or embarrassed. If the valence hypothesis was correct, then anesthetizing the right hemisphere should not have resulted in the recall of a negative type of emotion, such as feeling silly, stupid, depressed, sorry or embarrassed. Thus, we delayed publishing our results until we had figured out what we had actually discovered.

In April of 1991, I was invited to deliver a lecture on affective communication at a colloquium sponsored by the University of Texas at Austin. Also invited to speak at the colloquium was Ross Buck, Ph.D. (Professor of Psychology and Communication Sciences, University of Connecticut at Storrs), a social psychologist whose research interest included emotions, motivation and non-verbal behaviors [160,161,162]. We instantly realized that we had common research interests and I subsequently invited him to present a grand rounds lecture on emotions to the Department of Neurosciences, University of North Dakota School of Medicine, in Fargo, and to discuss potential collaborative research projects [1,163]. During his visit, I showed him the transcripts of the eleven subjects’ verbal recall of an emotional event before, during and after their right-sided Wada test. After looking through the transcripts and making copious notes, Ross concluded that almost all of the emotions recalled pre-Wada were primary emotional memories. During the right-sided Wada test, most patients minimized (decathected) their recall of the primary emotional memory and three patients actually denied experiencing a primary emotion. Four patients switched their recall of the event from a primary to a social emotion. One patient enhanced his social–emotional recall of the event and decathected the primary–emotional recall of the event. The last patient was complex. During his pre-Wada interview, he recalled meeting his son at the airport after the son was released from the penitentiary for multiple DWIs (driving while intoxicated). He (verbally) threatened to kill his son if he ever drove intoxicated again and began crying because he was “… sick of it. I was tired of it; I wasn’t going to stand for it no more” but denied feeling sadness. During the right-sided Wada, despite his overall flat affect when recalling the life event, he spoke with a discernably angry demeanor and remembered telling his son that he would kill him if he ever drove drunk again, but he did not break down and cry or verbally admit to feeling angry, sad or sick about the situation. This was classified as changing from a primary emotional recall (crying, sick of it) to another negative primary emotional recall (anger), but on reconsideration, it is most likely a primary emotional recall that was decathected even though he remembered verbally threatening to kill his son (Note: subsequent research has suggested that anger and other types of primary emotions can be classified as a social emotion under certain circumstances [164]). After Ross presented his assessment, my first reaction was to ask him what was a social emotion because in all my medical training and extensive readings of the behavioral neurology, neuropsychology and psychiatry literature, I had never come across the concept of social emotions. He explained that social emotions are learned beginning in late infancy and acquired in childhood through social interactions, whereas primary emotions are innate. After reading the relevant literature, we (Ross, Homan, Buck) agreed to publish the research under the title “Differential hemispheric lateralization of primary and social emotions: Implications for developing a comprehensive neurology for emotion, repression, and the subconscious” [5].

Emotions are feeling states that cannot be directly assessed or evaluated by researchers or clinicians [4,5,165,166,167]. Instead, one has to rely on emotional indicators to *infer* an emotional state. The indicators include autonomic and hypothalamic responses, such as changes in heart rate, respiration, lacrimation, sweating, pupillary size and capillary filling (blushing, blanching) and changes in neuroendocrine secretions, such as cortisol and norepinephrine, and somatic motor responses, such as arousal, freezing, fight–flight reactions and behaviors, such as laughing or crying, changes in non-verbal communication involving affective prosody, facial expressions and gestures, and changes in verbal–linguistic communication, such as verbally stating the depth and degree of one’s anger, depression or grief. However, from a neurologic perspective, inferring that a person is experiencing a specific emotion is not straight forward. For example, in brain-damaged patients with pathological regulation of affect, their laughing and crying is not indicative that they are actually experiencing a happy or sad feeling [157,168,169]. In fact, when queried, the patients will verbally report that their behaviors are socially embarrassing, unwanted, ego-alien and not under their volitional control. In contrast, if an emotional indicator is disrupted by a focal brain lesion, it does not imply that the patient cannot experience emotions inwardly. A good example are patients with motor aprosodia who still report being able to experience emotions, including depression, despite their affectively flat demeanor.

**Figure 4:**
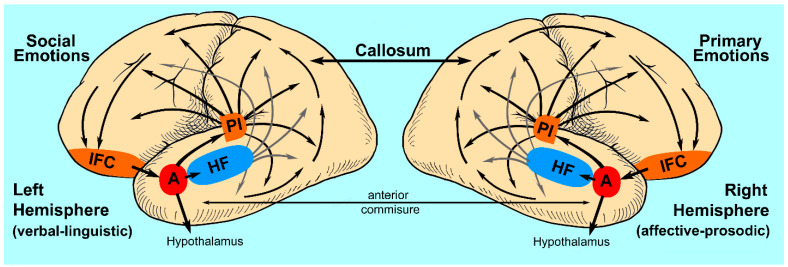
A schematized drawing depicting the emotional and mnestic pathways and neural networks that are involved with emotions, memory, executive functions and cognitive appraisal that ultimately furnish feedback to the amygdala and hippocampal formation (A = amygdala; HF = hippocampal formation; IFC = infero-frontal cortex; PI = posterior insula). Not shown are the ipsilateral parallel convergent inputs from the primary visual, somatosensory and auditory cortices that convey exteroceptive information to the amygdala and hippocampal [15]. The nodal point for processing exteroceptive information into memory is the hippocampal formation. However, the mnestic information is actually stored in the modality specific (unimodal) and higher-order (heteromodal) associational regions of the neocortex as a parallel distributed neural network (multiple gray arrows) [4,14,15,31,184]. This storage region is functionally equivalent to Wernicke’s ideational areas for propositional language and the right hemisphere’s equivalent for affective prosody (see Figure 1). The nodal point for generating an emotional reaction to exteroceptive information is the amygdala and its dense connections with the posterior insula. However, experiencing an emotion and storing it into memory is accomplished through a parallel distributed neural network (multiple black arrows) with the posterior insula serving as a nodal point in conjunction with and the unimodal and heteromodal associational regions of the neocortex [4,5,14,31,184]. The amygdala also has the capability of enhancing or diminishing the strength of factual memories stored in neocortex via its efferent connections to the hippocampal formation (short black arrows). The factual and emotional memories stored mainly in the posterior neocortices can, in turn, be relayed to the prefrontal neocortices for complex cognitive processing involving executive control of behavior (lateral pre-frontal areas) and cognitive appraisal of emotions (inferior and medial pre-frontal regions) that, in turn, can furnish feedback to the amygdala and hippocampal formation via efferent connections from the inferior frontal cortex. The emotional and mnestic information that is processed by each hemisphere can be shared via distributed connections that travel through the corpus callosum and anterior commissure. The temporal limbic system of each hemisphere has, at best, meager inter-hemispheric connections with each other. Figure reproduced from Ross, E.D. Differential hemispheric lateralization of emotions and related display behaviors: Emotion-type hypothesis. *Brain Sci*. **2021**, *11*, 1034, 2021 (Figure 1) [4] with permission.

Primary emotions (happiness, anger, sadness, fear, disgust and frightful surprise) and related displays, including affective prosody, are thought to be developmental derivatives of innate neonatal reflex behaviors to noxious or pleasant environmental stimuli, such as loud noises, bright lights, being fed or when parents interact using infant-directed speech characterized by excessive affective prosodic and facial expressions, also known as “motherese”, a universal behavioral phenomena underlying parental–infant communication [170,171,172]), or noxious or pleasant internal stimuli, such as thirst, hunger, colic, or when satiated [4]. These reflexive behaviors are organized in the brainstem since they may be observed in neonates who are born with anencephaly or severe hydranencephaly, a condition in which the entire forebrain and diencephalon may be missing [4,173,174,175,176]. Primary emotions develop as the infant begins to cognitively link their reflexive behaviors to specific external stimuli and events and acquire the cognitive ability to generalize emotional reactions to new situations [4]. For example, social referencing [177,178] begins at approximately one year of age when infants will look at a parent’s facial expression to help decide how to emotionally react to a new environmental stimulus, such as a stranger or a new inanimate object. Eventually, primary emotions (internal feeling state and related display behaviors) are instantiated in the forebrain as a lateralized right-hemisphere function, with the amygdala serving as the nodal point for experiencing a primary emotion as part of a widely distributed large-scale neural network (Figure 4) [4]. Primary emotions and related displays are universally recognized across cultures and, except for happiness, are negative in valence and associated with withdrawal, flight or fight types of responses [161,179,180,181,182,183].

Social emotions, such as envy, pity, embarrassment, guilt, jealousy, pride, shame, empathy and scorn, are acquired through social interactions beginning in late infancy and throughout childhood [4,5,160,182,183,185,186,187]. In infancy, the acquisition of social emotions is thought to be motivated by the biological drive for attachment. In later development, the acquisition of social emotions has been attributed to gaining the approval, affection and admiration of others and are sculpted by factors, such as societal, religious and educational expectations. Thus, social emotions and related displays are not universally recognized across cultures in contrast to the primary emotions. However, one should not conclude that social emotions are inferior drivers of behaviors because they are learned rather than innate. Just like the verbal–linguistic aspects of language, the brain is hard-wired to acquire social emotions but what is acquired is culturally dependent. Social emotions can be either positive or negative in valence. However, children learn how to manipulate their facial and vocal expressions (“display rules”) to meet cultural norms and to facilitate approach behaviors [160,161,187,188,189,190,191,192]. Thus, social emotions are associated with positive display behaviors that allows for amicable social interactions, even if the display behaviors are at odds with an individual’s internal emotional state. Display rules are accomplished by: (1) intensifying a felt emotional display, (2) dampening a felt emotional display, (3) masking (displaying no emotion when an emotion is felt), (4) simulating (displaying an emotion when no emotion is felt), (5) dissimulating (displaying a different emotion than the felt emotion), and (6) qualifying (displaying a different emotion on the upper versus lower face, i.e., a facial blend of emotion) [188,193,194,195]. It should be noted that recent research has also shown that facial blends can also occur across the left and right face, either as two distinct expressions, such as a frown–surprise or a smile–grimace or, more commonly, as a seemingly unitary expression that is the result of each hemisphere exerting independent contralateral motor control when subjects are shown emotion-provoking video clips [196]. This suggests that a facial expression, such as a smile, surprise, grimace or frown, can be the product of a comingled primary and social emotional reaction to an external event [4,196]. As a consequence of acquiring display rules, children also learn to cognitively manipulate their displays for deceitful purposes [193,197,198,199,200], an unfortunate human condition. Lastly, based on a series of patients with focal RBD and LBD, Buck and Duffy [160] established that display rules are a lateralized function of the left hemisphere, consistent with the concept that social emotions are a lateralized function of the left hemisphere [4,5].

## 4. Summary

As outlined in the manuscript, the deductive research establishing that affective prosody is a lateralized function of the right hemisphere and that the various aprosodic syndromes observed after focal RBD are functionally and anatomically analogous to the various aphasic syndromes observed after focal LBD was initiated by serendipitous clinical observations. While pursuing this line of research, once again, serendipitous clinical and unexpected research observations coupled with fortuitous academic collaborations lead to the development of a nascent neurology of depression and eventually the emotion-type hypothesis. My overarching goal for presenting the inductive research that initiated and guided the deductive research is three-fold: (1) to provide the reader with a more accurate and complete description of how the research actually occurred, (2) to provide constructs to help guide future research endeavors, and (3) to provide a better road map for young clinicians who are interested in pursuing a research career in behavioral neurology and related disciplines that also require an intellectually “prepared mind” to take advantage of serendipitous clinical encounters, unexpected research observations and fortuitous inter-disciplinary collaborations [1,2,3,4,6,7,201].

## Figures and Tables

**Table 1:** Classification of aphasic and aprosodic syndromes.

**APHASIAS**	**Spontaneous** **Speech**	**Verbal Repetition**	**Verbal Comprehension**	**Verbal Naming**
Motor	non-fluent	poor	good	poor ^§^
Sensory	fluent *	poor	poor	poor
Conduction	fluent	poor	good	poor
Global	non-fluent	poor	poor	poor
Transcortical Motor	non-fluent	good	good	poor ^§^
Transcortical Sensory	fluent	good	poor	poor
Mixed Transcortical	non-fluent	good	poor	poor
Anomic	fluent	good	good	poor
**APROSODIAS**	**Spontaneous** **Affective Prosody**	**Affective Prosodic Repetition**	**Affective Prosodic Comprehension**	**Facial Expression** **Naming**
Motor	poor	poor	good	good
Sensory	good	poor	poor	poor
Conduction	good	poor	good	poor
Global	poor	poor	poor	poor
Transcortical Motor	poor	good	good	good
Transcortical Sensory	good	good	poor	poor
Mixed Transcortical	poor	good	poor	poor
Agesic	good	good	good	poor

* Overall articulation is intact, but speech contains neologisms and related paraphasias making it, at times, incomprehensible. **^§^** Improves if given multiple choice options to bypass non-fluency issues.

## Data Availability

Not applicable.

## Glossary

**Fundamental frequency**	The lowest rate the vocal folds vibrate to produce a complex speech sound.
**Hertz**	An acoustic measure of the frequency of a complex speech sound in cycles per second that is usually the fundamental frequency. The first harmonic of a complex speech sound is usually one musical octave above the fundamental frequency or double the Hertz of the fundamental frequency. The second harmonic is usually one octave above the first harmonic or double the Hertz of the first harmonic. The third harmonic is double the Hertz of the second harmonic, etc.
**Intonation**	Manipulation of pitch over time
**Lexical tones**	Brief pitch contours associated with articulation of words in tone languages that are critical for word meaning.
**Operculum**	Region of the brain that surrounds the Sylvian fissure and overlies the insula.
**Pitch**	A psychoacoustic construct of how high or low a speech sound is perceived by a listener relative to a musical scale. Pitch is produced by vibrations of the vocal folds to produce a sound that has a fundamental frequency and various harmonics that are octave multiples.
**Semitones**	Converts Hertz to an acoustically logarithmic scale in which musical octaves (harmonics) are equally represented as a 12-semitone change in perceived pitch.
